# Pain Predictors in a Population of Temporomandibular Disorders Patients

**DOI:** 10.3390/jcm9020452

**Published:** 2020-02-06

**Authors:** Magdalena Osiewicz, Frank Lobbezoo, Bartosz Ciapała, Jolanta Pytko-Polończyk, Daniele Manfredini

**Affiliations:** 1 Department of Integrated Dentistry, Dental Institute, Faculty of Medicine, Jagiellonian University Medical College, 31155 Krakow, Poland; bartek.ciapala@uj.edu.pl (B.C.); jolanta.pytko-polonczyk@uj.edu.pl (J.P.-P.); 2 Department of Oral Kinesiology, Academic Centre for Dentistry Amsterdam (ACTA), University of Amsterdam and Vrije Universiteit Amsterdam, 1081LA Amsterdam, The Netherlands; f.lobbezoo@acta.nl; 3 School of Dentistry, University of Siena, 53100 Siena, Italy; daniele.manfredini75@gmail.com

**Keywords:** bruxism, temporomandibular disorders, depression, predictors, pain, patient population

## Abstract

The aim of the present study was to assess the potential role of some biological, psychological, and social factors to predict the presence of painful temporomandibular disorders (TMDs) in a TMD-patient population. The study sample consisted of 109 consecutive adult patients (81.7% females; mean age 33.2 ± 14.7 years) who were split into two groups based on Research Diagnostic Criteria for Temporomandibular Disorders (RDC/TMD) diagnoses: painful TMD and non-painful TMD. The presence of pain was adopted as the depended variable to be identified by the following independent variables (i.e., predictors): age, gender, bruxism, tooth wear, chewing gum, nail biting, perceived stress level, chronic pain-related impairment (GCPS), depression (DEP), and somatization (SOM). Single-variable logistic regression analysis showed a significant relationship between TMD pain and DEP with an odds ratio of 2.9. Building up a multiple variable model did not contribute to increase the predictive value of a TMD pain model related to the presence of depression. Findings from the present study supported the existence of a relationship between pain and depression in painful TMD patients. In the future, study designs should be improved by the adoption of the best available assessment approaches for each factor.

## 1. Introduction

Temporomandibular disorders (TMDs) are a heterogeneous group of conditions characterized by the presence of signs and symptoms such as pain in the masticatory muscles and/or the temporomandibular joint (TMJ), limited jaw movements, and TMJ sounds (i.e., clicking and/or crepitus) during function [1]. The Research Diagnostic Criteria for Temporomandibular Disorders (RDC/TMD; [2]) and their updated version, viz., Diagnostic Criteria for Temporomandibular Disorders (DC/TMD; [3]) are the current reference to standardize TMD diagnoses for clinical research purposes.

The protocol of (R)DC/TMD includes a dual axis evaluation, providing diagnoses based on both the physical data and the psychosocial data. The Axis I protocol is based on guidelines for oral history taking and clinical assessment, while the Axis II protocol encompasses an evaluation of several psychological factors (e.g., the level of pain-related impairment, depression, and somatization) that are considered key factors for TMD onset, clinical manifestation, and treatment outcome [4,5,6]. Additionally, the main Axis I diagnoses can be distinguished into two groups based on the presence or the absence of pain [3].

There are several biological, psychological, and social factors that may be associated with TMDs [1]. Amongst the possible biological etiological and risk factors, considerable attention has been given to bruxism, i.e., jaw-muscle activities occurring during sleep (identified as rhythmic or non-rhythmic) and/or wakefulness (characterized by repetitive or sustained tooth contact and/or by bracing or thrusting of the mandible), as well as to various oral habits, such as chewing gum and nail biting [7]. Those activities may cause painful TMDs due to overloading of the musculoskeletal structures, but evidence of a clear-cut association is lacking [8,9]. Similarly, the relationship between tooth wear and TMDs is worthy of further exploration [10]. Additionally, psychosocial status is also included among the possible risk factors for TMD [11,12,13,14]. In general terms, a shortcoming of the literature on the topic is that the majority of risk assessment studies on TMDs is based on non-patient populations [15,16], whilst the studies on TMD patient populations are focused on specific subpopulations of TMDs (e.g., muscle or joint disorders) [6,17].

Within these premises, given the heterogeneity of the conditions grouped under the umbrella term “temporomandibular disorders”, a possible improvement to current knowledge is to gain information on the separate painful and non-painful TMD diagnoses. Hence, the aim of the present study was to assess the potential role of the above factors to predict the presence of painful TMDs in a TMD-patient population, the null hypothesis being that they are not related to painful TMDs.

## 2. Materials and Methods

### 2.1. Participants

The study sample consisted of 109 consecutive adult patients (mean age 33.2 ± 14.7, range 18–72 years), 22 men (mean age 27.0 ± 7.5) and 87 women (mean age 34.6 ± 15.6), who sought for TMD treatment at the University Dental Clinic in Krakow, Poland and agreed to take part in the study. Criteria for inclusion in the study were: 1) the presence of one or multiple TMD diagnoses according to RDC/TMD; 2) the absence of signs and/or symptoms of systemic or local diseases possibly mimicking TMDs (e.g., fibromyalgia, hypothyroidism, lupus erythrocytes, scleroderma, Parkinson’s disease, Lyme disease, dystonia), as excluded by relevant consultants; and 3) the absence of other orofacial disorders possibly mimicking TMD-like signs and/or symptoms (e.g., neuropathic pain, tension type headaches, autonomic cephalalgias, migraines, psychogenic pain, myositis, infections, or any injuries), as excluded based on the patients’ history and relevant consultants’ reports.

The study was conducted in accordance with the principles of the Helsinki Declaration and approved by the Bioethics Committee of the Jagiellonian University (No. KBET/90/B/2010). All patients signed a written consent document.

### 2.2. Data Collection

All patients were examined by an investigator who had taken a specific training on RDC/TMD evaluation as well as on tooth wear assessment from a gold-standard examiner within the framework of a three-year specialty program in Orofacial Pain and Dysfunction [18]. All participants underwent a thorough assessment in accordance with the RDC/TMD guidelines to receive both Axis I and Axis II diagnoses based on the official Polish adaptation of the RDC/TMD [2,19]. Patients also filled out a comprehensive questionnaire that included, amongst others, the RDC/TMD questionnaire [2,19] and an oral habits questionnaire.

Collected data were managed as either outcome (i.e., dependent) or predictor (i.e., independent) variables to fit the study aim.

#### 2.2.1. Dependent Variables

The study sample was split into two groups based on RDC/TMD diagnoses:

1) Painful TMD group included patients with a diagnosis of myofascial pain/myofascial pain with limited opening (IA/IB) and/or arthralgia/osteoarthritis (IIIA/IIIB), independent of the presence of concurrent disc disorders diagnoses.

2) Non-painful TMD group included patients with disc displacement with reduction (IIA), disc displacement without reduction with limited opening/disc displacement without reduction without limited opening (IIB/IIC), and osteoarthrosis (IIIC), without any axis I group I or any other axis I group III diagnosis.

#### 2.2.2. Independent Variables—Biological Variables

1) Age (years) and gender were taken from each patient’s medical history provided before the visit.

2) The presence of bruxism was assessed using two questions from RDC/TMD questionnaire. Sleep bruxism was thus addressed with the question: “Have you been told, or did you notice yourself, that you grind your teeth or clench your jaws when you are asleep?” The presence of awake bruxism was assessed using the question: “Do you grind your teeth or clench your jaws during the day?”. As per suggestions from the consensus expert panel, at least one positive answer was considered indicative of possible bruxism (7).

3) Assessment of tooth wear was performed using the 5-point ordinal scale for the quantification of occlusal and incisal tooth wear proposed by Lobbezoo and Naeije [20]: 0 = no wear; 1 = visible wear within the enamel; 2 = visible wear with dentin exposure and loss of clinical crown height of ≤ 1⁄3; 3 = loss of crown height > 1⁄3 but < 2/3; and 4= loss of crown height ≥ 2⁄3.

4) Oral habits, such as chewing gum and nail biting, were assessed on the basis of a Yes/No answer.

#### 2.2.3. Independent Variables—Psychological Variables

1) The perceived stress level was assessed during the clinical investigation with the use of a 100 mm visual analog scale (VAS), where 0 mm = no stress and 100 mm = maximal stress.

2) The psychological status was assessed by means of the RDC/TMD Axis II, which comprises a psychosocial assessment based on three instruments. Chronic pain-related impairment was assessed based on Graded Chronic Pain Scale (GCPS) scores (0 – no disability, I – low disability/low intensity pain, II – low disability/high intensity pain, III – high disability/moderately limiting pain, and IV – high disability/severely limiting pain [21]. The study group included 8 patients with a depression (DEP) diagnosis made by a medical doctor prior to the study, which was confirmed by the RDC/TMD questionnaire. These patients had been taking antidepressants prescribed by their medical doctors.

Depression levels based on Depression Scale of the Symptoms CheckList-90R (SCL-90R) [22] might be either normal or moderate/severe [2]. Non-specific physical symptoms (viz., somatization) levels, based on the Somatization Scale (SOM) of the SCL-90R, were also identified as normal and moderate/severe. Summary of painful and non-painful TMD patients’ medication usage is shown in Table 1. None of the patients were under prolonged pharmacological treatment. In addition, patients were asked to avoid taking any medication for at least 24 h before the visit.

### 2.3. Statistical Analysis

The categorization into pain vs. non-pain was adopted as the dependent variable to be identified by the following independent variables (i.e., predictors): age, gender, bruxism, tooth wear, chewing gum, nail biting, perceived stress level, chronic pain-related impairment (GCPS), DEP, and somatization (SOM). All the independent variables were dichotomized. Dichotomization was based on the presence or the absence (i.e, yes/no) for GCPS, DEP, SOM, and bruxism. This was based on scores over or under the median value for numerical variables (i.e., age, perceived stress VAS scores, tooth wear).

Single-variable logistic regression analyses were performed to assess the association between the various predictors and the TMD group (i.e., pain group vs. non-pain group). To build a multi-variable regression model, an arbitrary threshold was set to include in the initial model all those factors that were significant at p<0.20 in the single-variable logistic regression analysis. Then, the variable with the weakest association was removed from the model until all the variables that were retained in the model showed a p<0.05. The odds ratios (OR) were assessed for each variable. OR values higher than 2 are commonly considered significant from a clinical viewpoint. Nagelkerke’s R-square was obtained as an estimation of the total variance explained by the predictor factors included in the model. All statistical procedures were elaborated with the R software, version 3.6.0 (Vienna, Austria).

## 3. Results

Regarding the marital status of the patients of the study group, 55.9% of them were never married while 37.6% were married. In total, 4.9% had been divorced, and 2% had been widowed. As far as the patients’ education is concerned, 67.9% were university graduates, 27.5% of the respondents had completed various secondary schools, and 4.6% had received only primary school education.

In total, 67.9% of the participants were diagnosed with at least one of the conditions belonging to the pain group, and the remaining 32.1% represented the non-pain group.

Single-variable logistic regression analysis showed a significant relationship between TMD pain and DEP with an odds ratio of 2.9 (Table 2). The other single regression models retrieving p-values lower than 0.2 were those with gender (OR = 0.4), bruxism (OR = 1.9), GCPS, and SOM (OR = 2.0).

The primary multiple logistic regression model including all the above-selected independent variables had DEP as the only significant variable (p-value <0.05). None of the other variables were significant, hence the model was not strong (R^2^ = 0.121). Stepwise elimination of variables led to a final model including just one significant variable (i.e., a single regression model with DEP as the independent variable). Among the other variables included in the primary multiple variable model (i.e., GCPS, SOM, bruxism, gender), none remained in the final model. Thus, building up a multiple variable model did not contribute to increase the predictive value of a TMD pain model related to the presence of depression. 

## 4. Discussion

The importance of biological and psychosocial risk factors for TMD patients is well-recognized in the literature [6,8,10,23,24,25,26], but knowledge is yet to be refined for the relationship of different factors with the various TMD diagnoses. In particular, since pain emerges as the main reason for patients to seek for treatment, information could be gathered on the factors that may predict the presence of pain in a population of TMD patients.

To our knowledge, the present investigation is the first study that tried to build possible TMD-pain prediction models with the evaluation of several factors (i.e., independent variables): age, gender, bruxism, tooth wear, chewing gum, nail biting, perceived stress level, chronic pain-related impairment, depression, and somatization. Based on the present study, the null hypothesis that none of the above factors are related to painful TMDs could be rejected. Despite the weakness of a significant relationship that prevented us from building predictable multiple variable models for TMD pain, single variable analysis gave some interesting suggestions. On one hand, it showed only a significant relationship between TMD pain and DEP, but on the other hand, it also showed chronic pain-related impairment and SOM scores; female gender and bruxism increased the chance, whilst male sex decreased the chance of belonging to the painful TMD group by approximately two times. Moreover, this investigation is the one of the first to assess risk factors in a population of TMD patients based on the presence of pain and not its location, in accordance with what was suggested for the evaluation of psychological factors in a previous paper [26]. Many studies on TMD risk assessment in the patient population are based on the inclusion of just one group of patients with a specific TMD diagnosis or the inclusion of patients with TMDs in general, i.e., independent of the specific signs and symptoms of TMDs [27,28,29,30,31]. It is also worth mentioning that, in this study, Axis I and Axis II diagnoses were made by an examiner who was thoroughly trained in the RDC/TMD protocol [18].

Based on literature studies, it is not surprising that depression is associated with TMD pain, since TMD-pain patients have been reported to have higher psychosocial impairment than TMD-free individuals [27,32]. According to Manfredini et al., both DEP and SOM scores have a significant relationship with GCPS rating [6,32]. Therefore, patients with the highest levels of pain-related disability were those with the highest levels of depression and somatization [17]. Thus, the inclusion of a higher number of chronic painful TMD patients might provide different results with respect to the present findings and should be considered in the design phase of future studies.

TMD pain is known as a complex disease being impacted by many disease-related variables in multiple domains, including biological, psychosocial, and pain processing variables [33]. Therefore, this patient population investigation supports the so-called biopsychosocial model of pain, which must include both an Axis I and Axis II evaluation [34]. In particular, building a prediction model to assess which factors predict the presence of pain-related TMD would be of help for the dentists while counseling a patient. It would also help limiting delays in interventions and/or unnecessary treatment, whilst the impact of psychological factors is huge.

There are several factors that might have affected the outcomes of this investigation. The main problem was that the regression model included factors that were not assessed based on the best available methods. For instance, awake and sleep bruxism were screened with the questions included in the history-taking questionnaire of the RDC/ TMD. Future studies should assess awake bruxism by means of an Ecological Momentary Assessment and sleep bruxism by means of instrumental measurement strategies such as electromyography [35,36]. Second, the adoption of the updated DC/TMD and the recruitment of a bigger sample size is a recommended strategy to further increase the usefulness of these data for cross-cultural comparison. The limitation of this investigation is the relatively small sample. This prevents us from satisfying a common rule of thumb for logistic regression analysis, postulating the need to include 10 observations with or without analyzed events per regression parameter [37]. Therefore, future studies should be based on a larger sample of patients with painful and non-painful TMDs. Moreover, this study was performed in a population of TMD patients without any control group of individuals without TMD signs or symptoms. Therefore, further studies should be performed with representative samples of patients with painful and non-painful TMD as well as with the non-TMD population. Finally, in order to achieve a proper evaluation of depression and somatization levels, the possibility to include a medical doctor as part of the team for the implementation of the psychological evaluation might be considered, as self-reported RDC/TMD Axis II scales do not provide any clinical diagnosis of psychopathology.

## 5. Conclusions

Findings from the present study supported the existence of a relationship between pain and depression in painful TMD patients. Such kind of analysis has never been performed in any previous studies, and it supports a need of considering both psychological factors and TMD signs and symptoms. In the future, study designs should be improved by the adoption of the best available assessment approaches for each factor.

## Figures and Tables

**Table 1:** Medications used by patients in painful and non-painful temporomandibular disorders (TMD) groups.

Medication	Painful TMD Patients	Non-painful TMD Patients
antihistamines	8.6%	21.4%
antibiotics	8.6%	14.3%
antidepressants	25.7%	7.1%
antiviral	8.6%	-
cardiological	11.43%	14.3%
contraceptives	5.7%	14.3%
proton pump inhibitors	11.4%	7.1%
painkillers	8.6%	-
others	11.4%	21.4

**Table 2:** Single regression models for the prediction of pain group patients. For each of the single regressions, the number of cases included in the analysis is shown. Associations are expressed as odds ratio (OR) and 95% confidence interval (CI).

Predictor Variable		Number	Single Regression				Multiple Regression (Nagelkerke R^2 ^= 0.121)			
			*p*-Value	OR	95% CI		*p*-Value	OR	95% CI	
**Age**		**109**	**0.398**	1.4	0.6	3.3				
stress		109	0.829	0.9	0.4	2.1				
DEP	0	63					0.019	2.2	0.8	6.2
					
	1	46	0.019	2.9	1.2	7.3				
GCPS	0	66					0.113	1.4	0.6	3.7
					
	1	43	0.113	2.0	0.9	4.9				
SOM	0	50					0.107	1.1	0.4	2.8
					
	1	59	0.107	2.0	0.9	4.5				
gender	F	89								
	M	20	0.063	0.4	0.1	1.1	0.063	0.5	0.2	1.4
bruxism	0	36								
	1	73	0.136	1.9	0.8	34.4	0.398	1.8	0.9	3.6
chewing gum	0	74								
	1	35	0.379	0.8	0.5	1.2				
nail biting	0	91								
	1	18	0.225	0.5	0.2	1.5				
tooth.wear		109	0.318	3.0	0.5	57.9				

moderate/severe depression (DEP); chronic pain-related impairment (GCPS); moderate/severe somatization (SOM).
